# Green finance, green development and decarbonization of the energy consumption structure

**DOI:** 10.1371/journal.pone.0300579

**Published:** 2024-04-05

**Authors:** Hui Zhu, Tianchu Feng, Xiaoliang Li

**Affiliations:** 1 Jiyang College, Zhejiang A&F University, Zhuji, Zhejiang, China; 2 School of Economics and Management, Zhejiang A&F University, Hangzhou, Zhejiang, China; Soochow University, CHINA

## Abstract

Energy plays a crucial role in global economic development, but it also contributes significantly to CO2 emissions. China has proposed a “dual-carbon” goal, and a key aspect to achieving this objective is finding effective ways to promote the decarbonization of the energy consumption structure (DECS). Compared with traditional finance, green finance is pivotal in advancing green and low-carbon development. However, the mechanism through which green finance impacts DECS has not been thoroughly explored. This study employs an enhanced weighted multi-dimensional vector angle method, which is more systematic and scientific, to measure DECS. Then, dynamic panel data from 30 provinces in China spanning the years 2003 to 2020 are used. A double fixed-effects model is applied to investigate the impact of green finance on the DECS and identify potential pathways. Results reveal that green finance significantly enhances DECS, primarily by reinforcing green development. The critical impact pathway involves the promotion of green technology innovation and green industry development. Moreover, the enhancing effect of green finance on the DECS is considerably significant in regions with relatively low government spending on science and technology (S&T), and where the focus is not on the “Atmospheric Ten” policy. The measurement of DECS is innovative, and the conclusions derived from it can offer compelling evidence for various social stakeholders. The government has the opportunity to establish a green financial system, supporting green technological innovation and the development of green industries. This approach can accelerate the DECS and work toward achieving the “double carbon” goal at an earlier date.

## 1. Introduction

A worldwide consensus exists on pursuing low-carbon green development considering the remarkable contribution of greenhouse gases (GHGs) to global warming. CO_2_ emissions are also acknowledged as a significant factor influencing China’s eco-environmental protection and constraining sustainable development [1]. An important problem lies in the fact that the energy consumption process is extremely highly carbonized. The high carbonization of the energy consumption structure is fundamentally determined by China’s natural resource reserves because energy consumption is derived from utilizing natural resources [2]. For China, this is reality is inescapable. Historically, China has been characterized as a “coal-rich, oil-poor, gas-poor” nation [3]. Traditional energy sources as essential strategic resources, such as coal, have played a crucial role in the extended process of economic development and industrialization. The *China Energy Development Report 2023* indicates that China’s total energy consumption, measured by standard coal, reached 5.41 billion tons in 2022, an increase of 2.9%. Among them, the proportion of coal consumption increased to 56.2%. This finding signifies that China’s reliance on traditional, highly carbonized energy sources for energy consumption remains high. Moreover, because of the high carbonization of conventional energy sources and inefficiencies in energy conversion that have yet to be adequately addressed, China, at this stage, continues to grapple with the dual challenges of natural resource waste and ecological environment destruction [4–6]. Balancing the costs of economic development and pollution control has become a pressing and practical issue that requires resolution [7]. In China, sustainable development has emerged as a national strategy for future economic growth [8, 9], aiming to achieve decarbonization of the energy consumption structure (DECS). The *14th Five-Year Plan for a Modern Energy System* emphasizes the imperative to “vigorously develop non-fossil energy sources,” while also highlighting the need to “strengthen energy conservation and carbon reduction with greater efforts.” Therefore, promoting the DECS has become a crucial method to achieve energy savings, consumption reduction, and advance green, low-carbon development for China [10, 11].

However, China, similar to many countries worldwide, is grappling with two significant challenges in transitioning toward low-carbon energy: lack of financial resource support and inefficient allocation of financial resources [12]. Traditional finance prioritizes economic development without considering ecological and environmental protection. Moreover, it inevitably amplifies the demand for traditional energy consumption in industrial development as it fosters an uptick in economic development, consequently contributing to a rise in CO_2_ emissions [13]. Contrary to traditional finance, green finance takes the protection of the environment as its primary goal and improves the efficiency of financial resource allocation. It is a crucial financial institutional arrangement for achieving sustainable development goals and fostering green transformation and development. Green finance is pivotal in realizing the DECS, providing specialized financial support. At the same time, green finance advocates using green energy while avoiding the misuse of traditional energy sources, such as coal [14–16]. Green finance also regulates the green development of the real economy, making a crucial contribution to pollution prevention and driving a low-carbon energy transition. However, compared with the “deep green” status of developed countries, China’s current stage of green development is still in the “light green” phase [17]. Hence, we raise the question of whether China’s green financial system at this stage can effectively promote the DECS. While existing studies have initiated preliminary discussions on the relationship between green finance and the DECS [3, 18], shortcomings still exist. First, the indicator measurement of DECS is relatively simple and cannot fully reflect reality. Second, the intrinsic influence mechanism of green finance on DECS has not been uncovered. Finally, further attention should be provided to heterogeneity analysis closely centered on the theme. We employ an enhanced weighted multidimensional vector angle method to measure DECS, investigate the effect of green on the DECS, and identify potential pathways.

This study provides three notable contributions. First, we employ an enhanced weighted multidimensional vector angle method to measure DECS, showcasing a more innovative approach. We utilize the method proposed by Li et al. [19], which not only addresses the base period selection issue but also provides the advantages of comparability across periods and samples, as well as increased applicability. This database can be used for empirically testing the relationship between green finance and DECS. Second, the research perspective is novel and unique. This study explores the effect of green finance on the DECS from the standpoint of green development, offering distinct empirical evidence. Green technology innovation (GTI) and green industry development (GID) were identified as key intrinsic impact mechanisms, overlooked in previous studies. Finally, in an in-depth exploration of the relationship between green finance and the DECS, the heterogeneous differences associated with government spending on science and technology (S&T) and the “Atmospheric Ten” policy are considered. The in-depth analysis is not only valuable for advancing reforms in the green financial system, but also provides inspiration for advancing policy development in DECS.

## 2. Literature review

Energy is very much relied upon by human activities, resulting in irrational energy consumption. Notably, industrialization has considerably intensified energy consumption and environmental pollution [4]. In terms of institutional arrangements, vertical fiscal imbalances induced by the fiscal system have fueled industrialization, leading to a substantial increase in energy consumption [20]. Moreover, this approach has significantly diminished energy efficiency and contributed to environmental pollution [1, 21, 22]. In addition to fiscal policy, expansionary monetary policy discourages renewable energy investment; thus, it does not contribute to the promotion of DECS [23]. Regarding transportation infrastructure, China’s highway transportation system plays a significant role in using traditional energy sources, such as gasoline and diesel [24]. From the perspective of digital economy development, the growth of the internet, including network information technology and the energy internet, has notably contributed to energy consumption [25]. Corporate digital transformation also enhances carbon productivity and achieves a certain level of DECS [26]. In the global context, geopolitical risk and economic uncertainty are also crucial factors affecting energy consumption [27]. Considering other factors, influences on energy consumption and use include technological innovation [28], environmental regulation [11], urbanization [29], and climate change [30]. In China, highly carbonized traditional energy sources constitute a significant portion of the energy consumption structure [3]. The key to promoting green development lies in the transition to low-carbon energy.

Traditional financial development may be detrimental to DECS. Some studies have shown that traditional financial development negatively affects renewable energy investment and usage [31]; it somehow exacerbates energy poverty [32]. However, green finance is a convergence point between finance and environmental protection. Song et al. [33] examined the effect of green finance on the efficient use of energy. Green credit, credit scale, environmental regulations, technological progress, and industrial structure are crucial in promoting efficient energy utilization. Liu et al. [17] conducted a study by constructing a dynamic stochastic general equilibrium model that includes the banking and insurance sectors. They found that combining insurance transition policies and green credit incentives effectively promotes a low-carbon energy transition.

The primary literature that is more relevant to the topic of this study are as follows. Lin et al. [34] employed the share of thermal power generation to measure the low-carbon transition in power generation and found the significant contribution of green finance. Gu et al. [3] explored the effect of green finance on energy consumption structure and its mechanisms. The study concludes that the improvement of energy consumption structure is significantly influenced by green finance, and this result is more evident in the central and western regions. However, they use the ratio of coal consumption to energy consumption to measure the DECS, a method deemed overly simple. By contrast, Chen and Bai [18] used a spatial spillover model and a threshold effect model to systematically investigate green finance, low-carbon energy transition, and environmental pollution. Their findings suggest that low-carbon energy transition is the primary mediating pathway for green finance to mitigate environmental pollution. However, the measurement method only calculates the level of DECS using a simple clip angle calculation. Both studies measure the DECS singularly, lacking systematicity and precision.

In summary, existing studies have extensively examined and discussed topics related to green finance and low-carbon energy transition. However, the following shortcomings still exist. First, the construction of the DECS is extremely simple. The measurement approach adopted by Gu et al. [3] and Chen and Bai [18] is not comprehensive and systematic. The present study measures the DECS using the enhanced weighted multidimensional vector angle method referred from Li et al. [19]. The method compensates well for the deficiencies in the above studies and has higher scientific rigor and rationality. Second, few studies have focused on the important role of green development. Chen and Bai [18] confirmed that environmental pollution is affected by green finance, and the main mechanism is the promotion of DECS. Gu et al. [3] also incorporated aspects of industrial structural transformation and energy efficiency in their study. However, theoretical analysis and empirical testing from the unique perspective of green development have not been conducted. In the present study, the perspective of green development is considered. Finally, previous studies have overlooked the significant influence of government spending on S&T and the “Atmospheric Ten” policy. Thus, we further consider the heterogeneous differences between these concepts. The limitations present an opportunity for the development of this study.

## 3. Hypothesis development

### 3.1. Green finance and DECS

Green finance actively promotes the DECS with its fundamental concept of advancing environmental protection. First, green finance provides investment and financing support for low-carbon energy projects [35]. For instance, it allocates special funds to enterprises, actively fostering the transition to low-carbon energy practices [36]. Second, green finance integrates environmental protection into the assessment process, altering fund allocation rules and reinforcing the fund matching function. Drawing on signaling theory, green finance sends a positive signal to the market, directing social capital toward the energy industry to advance green energy development and achieve the DECS [18]. Simultaneously, green finance enhances the policy-level expectations of market participants and boosts investor confidence in energy decarbonization. Green finance also mitigates the unpredictable risks associated with the DECS. By leveraging green identification mechanisms, such as information technology, green finance timely detects and manages social and environmental risks, reducing uncertainties through diversified investments [37, 38]. Finally, green finance provides incentives and regulatory initiatives to encourage the involvement of market players in green energy industries, such as photovoltaic and wind power [3], facilitating the gradual promotion of the DECS. Therefore, green finance significantly promotes the DECS. On the basis of this analysis, hypothesis 1 is drawn:

**Hypothesis 1:** Green finance has significantly contributed to the DECS.

### 3.2. Green finance, green development, and DECS

**Fig 1:**
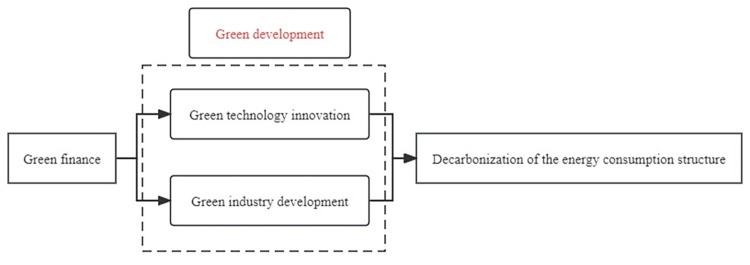
Impact mechanism of green finance on the DECS.

Traditional finance exhibits numerous shortcomings in promoting green development, and green finance serves to address these deficiencies. This study elucidates the role and pathway of green finance in decarbonizing the energy consumption structure from the perspective of green development (Fig 1).

The first pathway is green technology innovation (GTI). Green finance significantly promotes GTI [39], which is evident in the following aspects. First, efficiency improvement in financial resource allocation relies on green financial support, which is favorable for enhancing GTI. Innovation, characterized by uncertainty, high risk, and a lengthy cycle, requires dedicated financial support. Green finance reinforces allocating specialized resources for GTI, ensuring the smooth progression of related activities. Second, green finance can drive GTI by attracting talent concentration. Human capital constitutes the primary force behind innovation activities [40], and green finance presents a favorable environment for talent aggregation. GTI is pivotal for achieving DECS [17]. It facilitates the updating of technological processes and the formation of an effective synergy between new technologies and production processes. This approach not only promotes the enhanced utilization of green energy, but also reduces traditional energy consumption, thereby realizing DECS [41]. In addition, GTI minimizes the cost of energy use [42] and contributes to sustainable development by establishing new economic growth drivers through technological change.

The second pathway is green industry development (GID). Promoting GID is a prerequisite to genuinely achieve sustainable development [43]. Green finance mandates financial institutions to comprehensively consider environmental assessments when allocating financial resources and incorporate ecological environmental protection into the assessment process. This approach is conducive to the GID. Simultaneously, green finance sends positive signals to the market to attract social capital to the green industry. Therefore, under a robust green financial system, green industries can receive timely support from financial resources for long-term development. Moreover, green finance fulfils supervisory and management functions to effectively enhance the industrial greening of high-energy consumption and heavy-pollution industries. GID serves as a crucial driver for optimizing resource allocation, promoting the transformation of growth momentum and altering the energy consumption structure [44]. To a certain extent, GID can effectively reduce energy consumption and pollutant emissions, optimizing and safeguarding the ecological environment [45].

In summary, green finance significantly promotes the DECS by advancing green development paths, such as GTI and GID. On the basis of this analysis, hypotheses 2 and 3 are put forward:

**Hypothesis 2:** Green finance facilitates the DECS by fostering green technological innovation (InGTI).
**Hypothesis 3:** Green finance facilitates the DECS by fostering green industry development (lnGID).

## 4. Methods

### 4.1. Econometric model specification

The double fixed effects model proves advantageous in mitigating province and time-specific changes [46]. To keep the scientific validity of the results in the dynamic panel data, the double fixed effects model is used to test hypothesis 1, as follows:

$${DECS}_{i,t}=\alpha_{0}+\alpha_{1}{GF}_{i,t}+\alpha_{2}{Control}_{i,t}+\delta_{i}+\lambda_{t}+\varepsilon_{i,t},$$ (1)

where DECS is the dependent variable, and GF is the explanatory variable. Control represents some control variables that may affect the DECS. α_0_ represents the intercept term, and α_1_ and α_2_ represent the unknown regression coefficients. *i* denotes individual, and *t* denotes Year. δ_i_ and λ_t_ signify province-fixed effects and year-fixed effects, respectively, and ε_i,t_ represents the error terms.

To assess whether green finance influences the DECS by promoting green development, we construct model (2) for empirical testing.

$${GD}_{i,t}=\beta_{0}+\beta_{1}{GF}_{i,t}+\beta_{2}{Control}_{i,t}+\delta_{i}+\lambda_{t}+\varepsilon_{i,t}.$$ (2)

In the equation, GD indicate green development, the mediating variable, comprising primarily lnGTI and lnGID. β_0_ represents the intercept term, and β_1_ and β_2_ represent the unknown regression coefficients. Our primary focus lies in determining the significance of α_1_ and β_1_. The remaining variables remain consistent with the model.

### 4.2 Main variables

#### 4.2.1. Measures of decarbonization of the energy consumption structure

Previous studies commonly employ metrics, such as the share of coal energy consumption [3], the angle of entrainment measure [18], and CO_2_ emissions per unit of GDP [47] to gauge DECS. However, these indicators are often selected in isolation, lacking precision, representativeness, and systematicity. Consistent with the studies of Moore [48] and Li et al. [19], we assess the DECS using the enhanced weighted multidimensional vector angle method. The specific steps are discussed in the following section.

In the initial step, the provinces are arranged in order based on the magnitude of CO_2_ emissions generated by various types of energy sources, from the highest to the lowest. Assuming *n* types of energy, the consumption share $e_{t}^{j}(j=1,2,\ldots ,n)$ of each type is employed as a component of the spatial vector, enabling the construction of an *n*-dimensional spatial vector representing the energy structure. The spatial vector for the *t*th period is expressed as $E_{t}(e_{t}^{1},e_{t}^{2},\ldots ,e_{t}^{n})$.

In the second step, the baseline vector is selected. Assuming a theoretical lower limit on the degree of energy structure decarbonization exists, where all energy consumption originates from the type with the highest level of CO_2_ emissions, the *n*-dimensional spatial vector formed by the energy consumption structure in this scenario is established as the reference vector, denoted as E_0_(1,0,…,0). In this context, E_0_ represents the invariant and unique benchmark vector.

In the third step, the angle of the vector created by the change in the *j*th energy is computed. Using E_0_ as a reference, the value of the *j*th element of the E_0_ vector is substituted with the value of the *j*th element of E_t_ to form a new vector, denoted as E_a_. The angle between E_a_ and E_0_ is considered the angle of the vector exclusively formed by the alteration in the *j*th energy consumption structure. The vector E_a_ is represented as follows:

$$E_{a}=\left\{ \begin{array}{c} (e_{t}^{1},0,\ldots ,0)\;j=1\\ (1,0,0,\ldots ,0,e_{t}^{j},0,\ldots 0)\;j=2,3,\ldots ,n \end{array}\right.$$ (3)

At this point, the vector pinch angle θ_aj_ is obtained as follows:

$$\theta_{aj}=\left\{ \begin{array}{c} 0\;j=1\\ arccos\frac{1}{\sqrt{1+e_{t}^{j}}}\;j=2,3,\ldots n \end{array}\right.$$ (4)

Using E_t_ as the reference vector, the value of the *j*th component of vector E_t_ is substituted with the value of the *j*th element of E_0_ to form a new vector, denoted as E_b_. The angle between vectors E_b_ and E_t_ can likewise be understood as the vectors’ angle shaped by the change in the *j*th energy structure. The vector E_b_ is represented as follows:

$$E_{b}=\left\{ \begin{array}{c} (1,e_{t}^{2},\ldots ,e_{t}^{n})\;j=1\\ (e_{t}^{1},e_{t}^{2},\ldots ,e_{t}^{j-1},0,e_{t}^{j+1},\ldots ,e_{t}^{n})\;j=2,3,\ldots ,n \end{array}\right.$$ (5)

The angle θ_bj_ between the vectors E_b_ and E_t_ is calculated as follows:

$$\theta_{bj}=\left\{ \begin{array}{c} arccos\frac{e_{t}^{1}+\sum_{2}^{n}{\left(e_{t}^{j}\right)}^{2}}{\sqrt{1+\sum_{2}^{n}{\left(e_{t}^{j}\right)}^{2}}*\sqrt{\sum_{1}^{n}{\left(e_{t}^{j}\right)}^{2}}}\;j=1\\ arccos\frac{\sum_{1}^{j-1}{\left(e_{t}^{j}\right)}^{2}+\sum_{j+1}^{n}{\left(e_{t}^{j}\right)}^{2}}{\sqrt{\sum_{1}^{j-1}{\left(e_{t}^{j}\right)}^{2}+\sum_{j+1}^{n}{\left(e_{t}^{j}\right)}^{2}}*\sqrt{\sum_{1}^{n}{\left(e_{t}^{j}\right)}^{2}}}\;j=2,3,\ldots ,n \end{array}\right.$$ (6)

To avoid construction bias, the geometric mean of θ_aj_ and θ_bj_ is considered to obtain the angle of entrainment formed by the change in the energy mix in the *j*th, as follows:

$$\theta_{j}=\sqrt{\theta_{aj}\theta_{bj}}(j=1,2,\ldots ,n)$$ (7)

In the fourth step, the DECS is calculated by weighting the angles of the vectors formed by the changes in the energy mix of the *n* categories.

$$DECS=\sum_{i=1}^{n}\sum_{j=i}^{n}\theta_{j}$$ (8)

In the fifth step, all 20 categories of energy sources, as revealed in the China Energy Statistics Yearbook, were standardized to a common unit, namely, standard coal. Following these steps, China’s DECS was quantified. Contrary to Gu et al. [3] and Chen and Bai (2023) [18], the resulting DECS does not necessitate consideration of the base period selection problem. It holds the advantage of being comparable across various periods and samples, enhancing its overall applicability.

#### 4.2.2. Measures of green finance

The *Guiding Opinions on Building a Green Financial System* defines a green financial system as an institutional arrangement to promote the development of a green and transformed economy through financial instruments, such as green credit, green bonds, green development funds, and green insurance. Given the data availability, selecting corresponding indicators from green credit, green investment, green insurance, and green management is viable, as well as employing the entropy weight method to measure the green finance index [3, 49].

Among them, green credit is measured by the ratio of interest expenditure of the six energy-consuming industries to the total industrial interest expenditure. Green investment is measured by the ratio of investment in environmental pollution control to GDP. Green insurance is measured by the ratio of agricultural insurance income to the total agricultural output value. Green management is measured by the ratio of fiscal environmental protection expenditure to fiscal general budget expenditure.

#### 4.2.3. Measures of mediator variables

Two primary mediating variables characterize green development: (1) lnGTI, which is the mediator variable measured by the natural logarithm of the number of green patent applications in each province plus one. (2) lnGID, which is the mediator variable measured by the natural logarithm of the number of green industry enterprise branches in listed companies in each province plus one.

#### 4.2.4. Measures of control variables

This study also incorporates controls for a set of variables influencing DECS, primarily aimed at mitigating the adverse effects of potential omitted variables and ensuring the reliability of the results. Consistent with prior research, the study selects the following control variables: regional economic development (lnGDPPC), energy consumption (lnEnergy), technology market development (lnTec), urbanization degree (Urban), and fiscal pressure (FP).

lnGDPPC is gauged by the natural logarithm of regional GDP per capita. lnEnergy is quantified as the natural logarithm of energy consumption. lnTec is assessed by the natural logarithm of technology market turnover. Urban is determined by the urbanization rate. FP is measured by the difference between local fiscal expenditures and local fiscal revenues as a share of local fiscal revenues.

### 4.3. Sample and data

In this study, we employ balanced panel data spanning 30 provinces in China (excluding Tibet, Hong Kong, Macao, and Taiwan) from 2003 to 2020 for empirical analysis. The data sources are as follows: pertinent data for quantifying the DECS primarily originate from the China Energy Statistics Yearbook. Raw data for assessing green finance and additional data are sourced from the China Statistical Yearbook, the Statistical Yearbook of each province, and the China Insurance Yearbook. Data on green technology innovation are retrieved from the Chinese Research Data Services (CNRDS). Data on green industry development are obtained from the China Public Policy and Green Development Database (CPPGD). Data processing for this study is conducted using R software and Stata17 software.

Table 1 presents the outcomes of the descriptive analysis for the primary variables. Combining the examination of the maximum, minimum, and average values. Evidently, the median value of the DECS is merely half of the maximum, indicating a generally low level. Green finance is also at a low level. The development levels of green finance and DECS exhibit considerable variation among provinces, offering a foundation for further research. The median values of mediating variables, such as GTI (lnGTI) and GID (lnGE), are closer to the maximum values, but with a relatively high standard deviation, signifying an overall high level with substantial differences. The remaining control variables generally align with findings from prior studies. To mitigate the impact of multicollinearity, this study conducts a multicollinearity test. The test results indicate a mean variance inflation factor (VIF) of 3.59, with the degree of urbanization (Urban) registering a maximum value of 7.57. These findings suggest the absence of multicollinearity.

**Table 1:** Descriptive statistical analysis of variables.

Var	N	Max	Mean	Min	p50	SD
DECS	540	2.430	1.217	0.098	1.164	0.509
GF	540	0.839	0.153	0.042	0.126	0.101
lnGTI	540	10.486	6.638	1.099	6.752	1.734
lnGID	540	7.601	4.792	2.485	4.792	0.841
lnGDPPC	540	12.013	10.367	8.190	10.491	0.752
lnEnergy	540	10.642	9.232	6.528	9.269	0.732
lnTec	540	8.751	3.981	-1.661	3.959	1.907
Urban	540	0.896	0.541	0.265	0.528	0.145
FP	540	7.255	1.685	0.118	1.557	1.175

## 5. Empirical results

### 5.1. Baseline regression results

Table 2 demonstrates the basic findings for green finance and DECS. Column (1) displays the regression outcomes without including control variables, whereas column (2) showcases the results with all control variables included. In both columns, the coefficient of regression for core explanatory variable (GF) is notable, with a value of 3.177 in Column (1) and 1.137 in Column (2). Both values are statistically significant at the 1% level. This finding suggests a significant contribution of green finance to the DECS, thereby validating hypothesis 1.

**Table 2:** Baseline regression results.

	(1)	(2)
	DECS	DECS
GF	3.177***	1.137***
	(22.94)	(5.14)
lnGDPPC		0.071
		(1.05)
lnEnergy		-0.324***
		(-4.23)
lnTec		-0.033***
		(-2.71)
Urban		2.328***
		(5.31)
FP		0.028
		(1.18)
Province FE	Yes	Yes
Year FE	Yes	Yes
_cons	0.732***	1.949**
	(32.18)	(2.52)
R2	0.508	0.723
F	526.162***	55.200***
Obs	540	540

Note

*, **, and *** denote significance at 10%, 5%, and 1%, respectively. The *t* statistics are presented in parentheses, with the same conventions.

The control variables, lnEnergy, lnTec, and the degree of Urban demonstrated statistical significance. The regression coefficient performance of lnEnergy is significantly negative. At present, China is still dominated by high-carbon energy sources, such as coal [6], and the substitutability rate of low-carbon energy sources has not yet reached a high level. Therefore, more energy consumption may be accompanied by high carbonization of energy. The regression coefficient for technology market development (lnTec) exhibits a statistically significant negative performance. This finding could be attributed to the early technology market’s inclination to cater to heavily polluting industries, without a specific focus on the DECS. Moreover, achieving technological innovation for high-quality energy development is likely to entail a gradual process [28]. The regression coefficients for the degree of Urban demonstrate a significantly positive performance. With the advancement of urbanization, changes in the patterns of energy use are expected [50]. Urbanization facilitates the widespread adoption of modern energy by the public, promoting the use of low-carbon energy [51]. Consequently, urbanization is beneficial to the DECS.

### 5.2. Robustness checks

A series of robustness tests is conducted to ensure the reliability of the results (Table 3). First, an endogeneity test is conducted. In this study, green finance with one lag and two lags serves as an instrumental variable, and the system GMM method is employed for the endogeneity test. Following the test, the Cragg–Donald Wald F-value is 6411.205, significantly surpassing the threshold value of 10%. This finding indicates that the instrumental variable is robust. In addition, the *p*-value of Sargan’s test is 0.484, well above 0.1, rejecting the original hypothesis and confirming the absence of over-identification. The outcome in column (1) reveals that green finance continues to exhibit a positive and significant relationship, consistent with the original conclusion. Second, the potential effects of omitted variables are addressed. This study re-runs the regression after incorporating lnFDI and the proportion of secondary industry (IDU) into the control variables. The outcomes in column (2) remain consistent with supporting hypothesis 1. Third, the core variables are lagged by one period. Green finance (L.GF) lagged by one period in column (3) continues to exhibit positive and statistically significant results at the 1% level, signifying robust findings. Fourth, errors related to model specification are eliminated. In this study, the regression test is rerun using the OLS and RE model. Columns (4)–(5) present results consistent with the aforementioned findings.

**Table 3:** Robustness checks.

	(1)	(2)	(3)	(4)	(5)
	DECS	DECS	DECS	OLS	RE
GF	1.466***	1.298***	1.087***	1.760***	1.117***
	(7.92)	(5.76)	(4.51)	(7.10)	(5.72)
lnGDPPC	0.020	0.099	0.055	0.638***	0.089
	(0.39)	(1.22)	(0.78)	(7.15)	(1.34)
lnEnergy	-0.307***	-0.313***	-0.339***	-0.200***	-0.240***
	(-4.10)	(-3.89)	(-4.21)	(-6.31)	(-4.15)
lnTec	-0.016	-0.038***	-0.030**	-0.025*	-0.028**
	(-1.22)	(-3.07)	(-2.42)	(-1.77)	(-2.42)
Urban	3.520***	2.514***	2.535***	-0.246	1.954***
	(10.91)	(5.72)	(5.61)	(-0.78)	(5.61)
FP	0.072***	0.025	0.045*	0.066***	0.033
	(3.50)	(1.06)	(1.83)	(3.62)	(1.50)
lnFDI		-0.020			
		(-1.53)			
IDU		0.000			
		(0.18)			
Province FE	Yes	Yes	Yes	Yes	Yes
Year FE	Yes	Yes	Yes	Yes	Yes
_cons	1.656***	1.593*	2.163***	-3.309***	1.216*
	(3.35)	(1.79)	(2.61)	(-4.91)	(1.78)
R2	0.715	0.723	0.725	0.610	0.722
F/Wald chi2	185.303***	50.251***	54.780***	35.116***	1290.19***
Obs	480	540	510	540	540

### 5.3. Mechanism analysis

Table 4 presents the findings of the path examining the effect of green finance on DECS through the enhancement of green development. The effect of green finance on DECS is evident, displaying a significant positive relationship in Column (1). The result indicates that green finance enhances DECS significantly.

**Table 4:** Mechanism analysis.

	(1)	(2)	(3)
	DECS	lnGTI	lnGID
GF	1.137***	2.148***	1.731***
	(5.14)	(6.85)	(2.65)
lnGDPPC	0.071	-0.202**	0.128
	(1.05)	(-2.09)	(0.64)
lnEnergy	-0.324***	0.941***	-0.542**
	(-4.23)	(8.68)	(-2.40)
lnTec	-0.033***	0.063***	0.085**
	(-2.71)	(3.65)	(2.38)
Urban	2.328***	4.793***	-0.041
	(5.31)	(7.71)	(-0.03)
FP	0.028	-0.066**	-0.220***
	(1.18)	(-1.98)	(-3.17)
Province FE	Yes	Yes	Yes
Year FE	Yes	Yes	Yes
_cons	1.949**	-3.972***	8.784***
	(2.52)	(-3.62)	(3.84)
R2	0.723	0.973	0.388
F	55.200***	773.904***	13.408***
Obs	540	540	540

The results in Column (2) illustrate the effect of green finance on GTI. GF’ regression coefficient is 2.148, which is significant at the 1% level, signifying that green finance effectively promotes GTI. Existing studies suggest that GTI is a crucial driver of green economic development [52, 53]. This idea is attributed to the fact that GTI contributes to expanding the scale of green energy use and improving utilization efficiency [54]. For example, increasing the proportion of energy derived from sources, such as hydropower and wind energy, can lead to reduced CO_2_ emissions [55, 56]. Consequently, GTI plays a pivotal role in promoting DECS, contributing to the mitigation of CO_2_ emissions and the alleviation of environmental pollution [57]. This analysis demonstrates that green finance facilitates DECS by fostering GTI, confirming Hypothesis 2.

Column (3) displays the results concerning the effect of green finance on GID. The findings reveal that the regression coefficient of green finance is 1.731, which is significant at the 1% level, indicating its substantial contribution to GID. The green industry, as the focal point of sustainable development strategy implementation, is crucial in ensuring and optimizing ecological and environmental protection [45]. Some studies have indicated that the promotional impact of GID on green energy is evident in the manufacturing industry and micro-enterprises [58, 59]. Over time, GID expands the scope and depth of green energy utilization, fostering DECS [60]. In summary, green finance facilitates DECS by promoting GID, validating Hypothesis 3.

## 6. Heterogeneity analysis

To delve more deeply into the external factors influencing the effect of green finance on DECS, the perspectives of government spending on S&T and the “Atmospheric Ten” policy are considered.

### 6.1. Government spending on S&T

Substantial endowment differences are evident among different regions, reflecting divergent development trajectories [43]. Local governments exhibit significant variations in government spending on S&T due to fiscal considerations [61]. Nonetheless, this condition influences energy utilization efficiency [22]. Studies have indicated that government spending on S&T plays a pivotal role in promoting the development of the energy industry [62]. In regions with lower spending, green finance is more likely to offer support for DECS, compensating for the shortfall. This study anticipates that the promotional impact of green finance on DECS may be more pronounced in regions with lower government spending on S&T.

To validate this perspective, the average value of government spending on S&T is used as the division criterion. The sample is divided into a lower government spending on S&T group (Low S&T) and a higher government spending on S&T group (High S&T), and empirical tests are conducted using Model (1). Column (1) of Table 5 presents the results for the sample with low S&T, whereas column (2) shows the results for the sample with high S&T. The regression coefficient of green finance in column (1) is 1.594, statistically significant at the 5% level. However, that in column (2) is 0.357, which is not statistically significant. These results indicate that the effect of green finance on DECS is more pronounced in the sample with low S&T than in the sample with high S&T, consistent with the anticipated outcomes.

**Table 5:** Heterogeneity analysis.

	(1)	(2)	(3)	(4)
	Low S&T	High S&T	Non-Policy	Policy
GF	1.594**	0.357	2.544***	-0.643
	(2.10)	(1.25)	(6.49)	(-0.91)
lnGDPPC	0.197**	-0.272**	0.122	-0.048
	(2.05)	(-2.08)	(1.55)	(-0.26)
lnEnergy	-0.216**	-1.297***	-0.310***	0.597
	(-2.45)	(-4.50)	(-3.91)	(0.97)
lnTec	-0.048***	0.034	-0.041***	-0.059
	(-3.46)	(0.84)	(-3.21)	(-0.97)
Urban	1.005	4.958***	1.598***	1.194
	(1.52)	(6.79)	(2.74)	(0.64)
FP	0.051*	0.016	0.032	0.034
	(1.93)	(0.22)	(1.33)	(0.19)
Province FE	Yes	Yes	Yes	Yes
Year FE	Yes	Yes	Yes	Yes
_cons	0.367	12.432***	1.582*	-3.988
	(0.39)	(5.10)	(1.89)	(-0.80)
R2	0.603	0.858	0.706	0.745
F	22.713***	27.390***	44.946***	8.762***
Obs	396	144	484	56

### 6.2. “Atmospheric Ten” policy

In September 2013, the State Council issued the *Air Pollution Prevention and Control Action Plan*, commonly known as the “Atmospheric Ten” policy. This policy outlines 45 essential tasks, with energy restructuring being a crucial component. For instance, policies such as “accelerating the adjustment of energy structure” and “controlling the total amount of coal consumption” are designed to advance DECS effectively. Specifically, the government has imposed stringent requirements on regions, such as Beijing–Tianjin–Hebei (BTH), the Yangtze River Delta (YRD), and the Pearl River Delta (PRD), to reduce total coal consumption. In the “Atmospheric Ten” policy, the BTH, YRD, and PRD regions are focal points for government attention. Following the policy’s implementation, these key regions are expected to undertake governance initiatives guided by the policy directives, including the implementation of adjustments to the energy industry’s low carbonization. DECS in these regions are likely to be influenced by the “Atmosphere Ten” policy. This study anticipates that the promoting effect of green finance on DECS may be more pronounced in regions that are not prioritized in policy considerations.

To validate this perspective, the regions of BTH, YRD, and PRD are designated as the policy focus implementation group (Policy), following the promulgation and implementation of the policy. The remaining regions are classified as the control group (Non-policy). Empirical tests are conducted using Model (1) for each group. Column (3) in Table 5 demonstrates the results for the sample of regions not focusing on “Atmosphere Ten” policy, and column (4) displays the results for the sample of “Atmosphere Ten” policy focus regions. The regression coefficient of green finance in Column (3) is 2.544, which is statistically significant at the 1%. However, the regression coefficient of green finance in Column (4) is −0.643, which is not statistically significant. The results above indicate that the promotional effect of green finance on DECS is more pronounced in non-policy priority regions than in policy priority regions, consistent with the expected outcomes.

## 7. Conclusion and policy implications

Effectively promoting DECS is crucial for achieving sustainable development in the future. Currently, green finance plays a dual role by providing specialized financial resources for the high-quality development of energy industries and reinforcing environmental regulations. Therefore, actively exploring how green finance can effectively advance DECS is a significant practical challenge in realizing sustainable development goals. This study empirically analyzes dynamic panel data from 30 provinces in China, spanning from 2003 to 2020, to explore the effect of green finance on DECS and identify potential pathways. The study reveals that green finance significantly enhances DECS, primarily by reinforcing green development. The critical impact pathway involves the promotion of GTI and GID. Furthermore, the enhancing effect of green finance on DECS is more significant in regions with lower government spending on S&T, and where the focus is not on the “Atmospheric Ten” policy. Compared with the studies of Gu et al. [3] and Chen and Bai [18], the present study employs an enhanced weighted multidimensional vector angle method to measure DECS, achieving a more systematic and scientific approach. Furthermore, the intrinsic mechanism of green finance affecting DECS was revealed in this study, bridging existing gaps. Meanwhile, the proposed heterogeneity analysis has some uniqueness and novelty.

The findings hold significant practical and theoretical importance. Building on these conclusions, this study puts forth pertinent policy recommendations. First, governments should recognize the key role of green finance in achieving DECS. On one hand, they should formulate robust green finance regulations, enhance the supervision of financial institutions, and standardize market behavior. On the other hand, they should support the development of markets, such as green credit and green bonds, to provide financial liquidity to society, thereby promoting DECS.

Second, the government should actively promote green technology innovation and green industry development to enhance DECS. For heavily polluting industries, the government can introduce special green funding policies for sewage, energy-saving, and other environmental protection technologies to encourage the greening of industries. For environmental protection and other green industries, the government can provide a platform to facilitate the sharing of talents and resources within the industry, promoting the development of the green industry. Such platforms can take the form of online platforms or a combination of various forms, including offline meetings. The ultimate goal is to realize DECS.

Third, governments can pursue DECS through various forms of policy mix. They can use green finance as the foundation for multiple forms of policy mix. For instance, in areas with low S&T or non-policy, green finance can serve as a potent complementary tool to support DECS. Simultaneously, higher-level governments should focus on the energy industry in disadvantaged regions and explore suitable development models based on local conditions.

This study examines whether green finance promotes DECS and analyzes the significant role of green development, offering a valuable reference for enhancing green finance. Only front exploration using China’s provincial panel data has been conducted because of data accessibility challenges, and an in-depth analysis at the city and enterprise levels has not been performed. Future research could employ Python software to textually analyze the annual reports of firms, enabling the extraction of firm data for measuring DECS. Expanding research efforts related to DECS from the firm level may yield new findings.

## Supporting information

S1 Data (XLSX)
